# Bilateral *Pseudomonas* endophthalmitis after bilateral simultaneous cataract surgery: *primum non-nocere*

**DOI:** 10.5935/0004-2749.20220097

**Published:** 2025-08-21

**Authors:** Eugene R. Ting, Julie A. Trethowan, Tyler R. Blah, Michael Lin, Michael Kvopka, Brendon Lee, Tammy Y. Cai, Jessica X.L. Li, Michele Y. Fu, Declan T. Lloyd, Ru Min Ong, Oscar A. Boag Taylor, Nicholas Xiradis, Amitouj S. Sidhu, Ashish Agar, Ian C. Francis

**Affiliations:** 1 University of Sydney, Australia; 2 University of New South Wales, Sydney, Australia; 3 Department of Ophthalmology, Prince of Wales Hospital, Sidney, Australia; 4 University of Notre Dame, Sydney, Australia

Dear Editor:

The authors read Arshinoff et al.’s^(1)^ response that voiced concerns about the authors’ reply regarding bilateral postoperative endophthalmitis (POE), and subsequent eye removal in patients following bilateral simultaneous cataract surgery (BSCS).

Indeed, the authors agree on the requirement to adhere to the publication ethics. These are standards of academic integrity that physicians aspire to. The authors would extrapolate their concern to facing the consequences of bilateral POE and subsequent eye loss following BSCS, a consequence that can be prevented by nonsimultaneous cataract surgery.

However, whether to perform BSCS remains controversial. The White Paper (2006) has been superseded over the last 15 years. It cannot currently be justified as having a “resolved issue”, or representing the contemporary standard of ophthalmological practice, as highlighted by several salient points below.

Firstly, phacoemulsification surgeons often describe “leaking wounds”. Arguably, wounds that allow postoperative aqueous humor leakage can decrease raised intraocular pressure. However, the authors have published over 25 Medline articles demonstrating leaking wounds, permitting extraocular pathogens entry^(2)^. As the eye is bacteria-free after the uneventful cataract surgery, it is the later entry of pathogens that results in POE^(3)^. The relationship between nonclosed corneal incisions and leaking wounds is best described by an eminent academic Australian ophthalmologist, who indicated that the largest bacteria are smaller than the smallest corneal wounds^(2)^.

Secondly, the White Paper refers to Seidel testing to confirm wound integrity. However, it refers only to aqueous humor leaking from the eye and not extraocular fluid leakage. The failure to precisely document Seidel’s testing technique and the importance of explicit incision compression do not exclude extraocular fluid leakage.

Thirdly, stromal hydration is performed by most surgeons to ensure clear corneal incision closure. This is not done to prevent aqueous humor from leaking from the eye but to prevent extraocular fluid from entering the eye. Studies have shown that wound integrity duration can be anything from just 30 minutes to 5 hours^(4)^. This may well explain “the plethora of recent reports regarding increased rates for postoperative infection”, as per the White Paper. Notably, the authors’ own country, Australia, documented the highest POE rate (0.834%) in the world in 2009.

Despite advances in POE treatment, the best-corrected visual acuity of patients with bilateral evisceration because of POE remains extremely poor. The fact that Intelligent Research in Sight (IRIS) in the USA states that some patients with POE achieve good visual outcomes (VOs) of 20/20 is not certainly a normal feature. The authors’ own publications show that reasonable VOs in large cohorts are 92.3%, achieving 20/12 at 1 month^(5)^.

Arshinoff et al.^(1)^ stated that the authors did not cite the European Society of Cataract & Refractive Surgeons study. However, the authors have several publications already addressing this issue^(6)^.

The unprecedentedly low POE rate (0.007%, n=14,352), quoted by professor Arshinoff, is noteworthy. The authors would be pleased to assess the relevant unreferenced source document and determine whether this study was prospective, consecutive, no-exclusions, singleor multi-surgeon, reported wound closure or nonclosure and demonstrated 1 month of postoperative follow-up.

Encouragingly, the Hippocratic Oath remains at the foremost. However, the authors were perplexed by Arshinoff et al.’s^(1)^ statement that the authors suggested that BSCS was intended to create bilateral simultaneous endophthalmitis. The authors reject this argument but highlight that BSCS directly facilitates the conditions in which bilateral POE occurs.

The authors remain concerned about BSCS inherent risks, as are most regulatory bodies, including Royal College of Ophthalmologists, American Academy of Ophthalmology, Canadian Ophthalmological Society, and Royal Australian and New Zealand College of Ophthalmologists.

The suggestion that the authors’ concerns are “simply nonsense” seems to unintentionally oppose Arshinoff et al.’s^(1)^ subsequent reference to the Medical Board of Australia’s request that we “treat each other with respect”.The authors appreciate life changes, as Arshinoff et al.^(1)^ highlighted, in creating an analogy between BSCS and motor vehicle accidents (MVAs). However, as Hippocrates would have undoubtedly confirmed, whether speaking Greek or Latin, any MVA fatality is an involuntary tragedy. Blinding complications from an elective surgical procedure represent a totally avoidable issue.
